# Combined assessment of 25(OH)D, CD4^+^/CD8^+^ ratio, and prognostic nutritional index for anti-dsDNA-based stratification in systemic lupus erythematosus

**DOI:** 10.3389/fimmu.2026.1841620

**Published:** 2026-04-29

**Authors:** Chaoju Yang, Juntao Meng, Nan Ding, Yanqing Tie

**Affiliations:** 1 Department of Clinical Laboratory, Hebei General Hospital, Shijiazhuang, Hebei, China; 2 Hebei Key Laboratory of Molecular Medicine, Shijiazhuang, Hebei, China; 3 Hebei Clinical Research Center for Laboratory Medicine, Shijiazhuang, Hebei, China

**Keywords:** 25-hydroxyvitamin D, anti-dsDNA antibody, CD4+/CD8+ ratio, prognostic nutritional index, systemic lupus erythematosus

## Abstract

**Background:**

Systemic lupus erythematosus (SLE) is a heterogeneous autoimmune disease in which anti-double-stranded DNA (anti-dsDNA) antibodies are closely associated with disease activity and organ involvement. However, the relationships of 25-hydroxyvitamin D [25(OH)D], CD4^+^/CD8^+^ ratio, and prognostic nutritional index (PNI) with anti-dsDNA antibody status and titer stratification remain incompletely understood.

**Methods:**

A total of 126 patients with SLE were enrolled and classified into an anti-dsDNA-negative group and an anti-dsDNA-positive group. According to anti-dsDNA titers, patients were further stratified into low-titer and high-titer groups. Clinical characteristics and laboratory parameters, including 25(OH)D, CD4^+^/CD8^+^ ratio, and PNI, were compared among groups. Receiver operating characteristic curve analysis was performed to evaluate the discriminatory performance of 25(OH)D, CD4^+^/CD8^+^ ratio, PNI, and their combined model.

**Results:**

Compared with anti-dsDNA-negative patients, anti-dsDNA-positive patients had significantly lower levels of C3, C4, the CD4^+^/CD8^+^ ratio, 25(OH)D, and PNI, together with a higher prevalence of renal involvement (all *P* < 0.05). Significant differences in 25(OH)D, the CD4^+^/CD8^+^ ratio, and PNI were also observed across the anti-dsDNA-negative, low-positive, and high-positive groups. In ROC analysis, 25(OH)D showed the highest AUC among the individual biomarkers for anti-dsDNA serostatus, whereas the combined assessment achieved the highest overall AUC. For stratifying low-positive from high-positive anti-dsDNA titers, the combined assessment showed better discriminatory performance than PNI and C3.

**Conclusions:**

Combined assessment of 25(OH)D, the CD4^+^/CD8^+^ ratio, and PNI may provide complementary value for anti-dsDNA-based stratification in SLE. Its performance was comparable to C3 for anti-dsDNA serostatus and superior to C3 for stratifying low-from high-positive anti-dsDNA titers. These findings should be considered exploratory and require validation in external cohorts.

## 1 Introduction

Systemic lupus erythematosus (SLE) is a chronic autoimmune disease characterized primarily by immune dysregulation. Its pathogenesis is complex and is closely associated with multiple factors, including genetic susceptibility, environmental exposure, immune imbalance, and nutritional status (1–3). SLE presents with marked clinical heterogeneity and a relapsing–remitting course, which seriously affects patients’ quality of life and may even be life-threatening. Therefore, early and accurate assessment of disease status and clear disease stratification are of great importance for optimizing treatment strategies and improving patient prognosis (4, 5).

Anti-double-stranded DNA (anti-dsDNA) antibody is one of the highly specific autoantibodies in SLE. Its titer is closely associated with disease activity and severe complications such as renal involvement, and it is considered a key biomarker for clinical diagnosis, disease severity assessment, and treatment monitoring (6, 7). At present, anti-dsDNA antibodies are mainly detected by enzyme-linked immunosorbent assay and indirect immunofluorescence assay in clinical practice. However, these methods are not widely available in primary care hospitals, which limits their utility for rapid clinical stratification and early intervention. Therefore, identifying simple and readily accessible auxiliary indicators to help assess anti-dsDNA antibody status may have important clinical value (8).

The prognostic nutritional index (PNI), which is calculated from serum albumin levels and lymphocyte counts, is a simple indicator for nutritional and immune assessment. It can reflect both nutritional status and immune reserve function. Because it is easy to obtain and relatively low in cost, PNI has been widely used for prognostic evaluation and disease stratification in a variety of conditions, including autoimmune diseases and malignancies (9–11). As key components of the immune system, lymphocyte subsets, particularly CD4+ T lymphocytes, CD8+ T lymphocytes, and their ratio (CD4^+^/CD8^+^), directly reflect immune balance. Since immune dysregulation is a central mechanism in the development of SLE, abnormalities in the CD4^+^/CD8^+^ ratio have been reported to be closely associated with disease activity and autoantibody production in SLE (12, 13).

25-hydroxyvitamin D [25(OH)D] is a stable marker of vitamin D status in the body. In addition to its well-known role in calcium and phosphorus metabolism, it also has important immunomodulatory functions. It may participate in the development of immune dysregulation in SLE by regulating T-cell differentiation, suppressing the activation of autoreactive lymphocytes, and reducing the release of inflammatory cytokines (14, 15). Previous studies have shown that low 25(OH)D levels are closely associated with higher disease activity, abnormal autoantibody profiles, and an increased risk of organ damage in patients with SLE, suggesting that it may serve as a simple potential marker for evaluating immune status and disease severity (16, 17).

At present, studies on the relationships of PNI, 25(OH)D, and CD4^+^/CD8^+^ ratio with anti-dsDNA antibodies in patients with SLE remain limited. Most previous studies have mainly focused on the association between a single marker and disease activity, while few have simultaneously evaluated the diagnostic performance of PNI, 25(OH)D, and CD4^+^/CD8^+^ ratio for both the qualitative status of anti-dsDNA antibodies (positive vs. negative) and titer stratification (low titer vs. high titer). In addition, most existing studies have emphasized risk factor analysis rather than a systematic assessment of diagnostic value. Therefore, the present study enrolled patients with SLE and focused on three simple and readily available blood-based indicators, namely PNI, 25(OH)D, and CD4^+^/CD8^+^ ratio. Receiver operating characteristic (ROC) curve analysis was used to evaluate their value in stratifying anti-dsDNA antibody positivity and in stratifying antibody titers. At the same time, by comparing baseline characteristics among the three groups, we further explored their associations with anti-dsDNA antibody status. Our findings may provide a useful reference for rapid clinical assessment of antibody levels and offer new insights into disease stratification and individualized intervention in SLE.

## 2 Materials and methods

### 2.1 Study population

**Figure 1:**
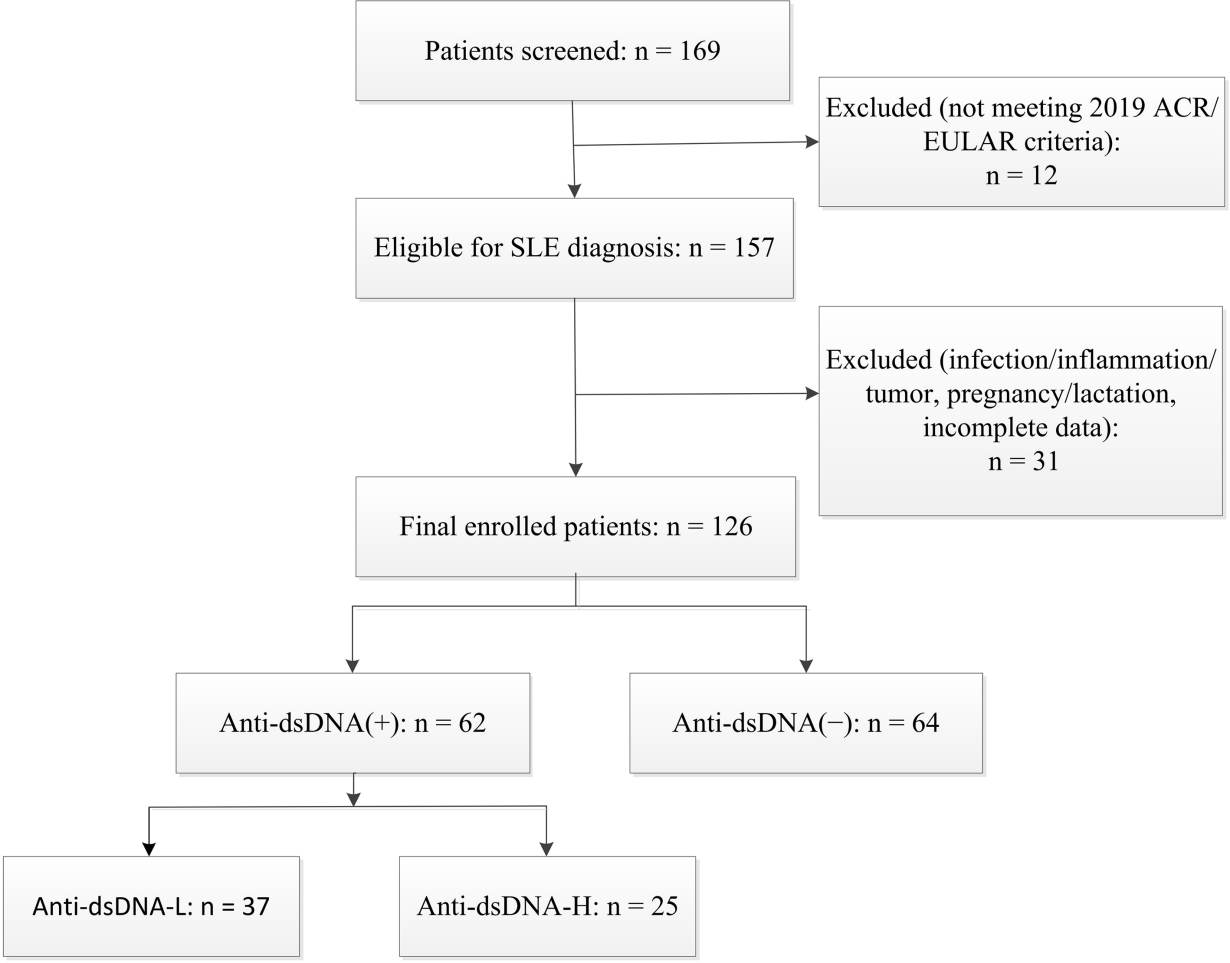
Flow diagram of patient screening, exclusions, and final inclusion.

This single-center, hospital-based cross-sectional study was conducted at Hebei General Hospital (Shijiazhuang, China) between December 2023 and December 2024. Consecutive hospitalized patients with established SLE were enrolled, including both newly diagnosed and previously diagnosed cases who were admitted for disease evaluation, flare management, or treatment of complications. A total of 126 patients with SLE were enrolled. All laboratory measurements were obtained within 24 hours after admission. Patients were classified into an anti-dsDNA-negative group [anti-dsDNA(-)] and an anti-dsDNA-positive group [anti-dsDNA(+)]. The anti-dsDNA(+) group was further divided into low-titer [anti-dsDNA-L] and high-titer [anti-dsDNA-H] subgroups according to antibody titers. A flow diagram of patient screening, exclusions, and final enrollment is shown in **Figure 1**.

### 2.2 Diagnostic criteria

SLE was classified using the 2019 ACR/EULAR criteria. hematologic involvement refers to the 2019 EULAR/ACRSLE classification standards, and is based on the reduction of white blood cells, lymphocytes, or platelets; renal involvement refers to the 2019 EULAR/ACRSLE classification standards and the Chinese Lupus Nephritis Diagnostic Guidelines, and is based on proteinuria, abnormal renal function, or lupus nephritis confirmed by renal biopsy (18).

### 2.3 Inclusion and exclusion criteria

Eligible participants diagnosed with SLE were hospitalized for initial evaluation and treatment, had complete clinical and laboratory data. Patients were excluded if they had acute or chronic infections or other inflammatory diseases, were pregnant or breastfeeding, or had incomplete clinical records.

### 2.4 laboratory measurements

All laboratory data were extracted from inpatient medical records and archived laboratory reports. All tests were performed in the Department of Clinical Laboratory of Hebei General Hospital according to standard operating procedures, with routine internal quality control and regular instrument calibration. Neutrophil count, lymphocyte count, and platelet count were measured using a Sysmex automated hematology analyzer. ESR was determined by the modified Westergren method. Serum ALB, C3, C4, and CRP were measured using an automated biochemical analyzer, with ALB assessed by the bromocresol green method. Lymphocyte subsets were analyzed by flow cytometry using a Beckman Coulter flow cytometer after direct immunofluorescence staining. Serum 25(OH)D was measured by electrochemiluminescence on a Roche E602 analyzer. Anti-Sm, anti-SSA, and anti-SSB antibodies were detected by chemiluminescence immunoassay using an iFlash automated analyzer (YHLO, China). Anti-dsDNA antibodies were measured using the EUROIMMUN *Crithidia luciliae* indirect immunofluorescence test. Results were classified as negative, 1:10, 1:32, 1:100, 1:320, and >1:320, with negativity defined as <1:10 and positivity beginning at 1:10. For analytical purposes, titers of 1:10 and 1:32 were grouped as the low-positive titer range, whereas titers ≥1:100 were grouped as the high-positive titer range. PNI was calculated as ALB (g/L) + 5 × LYMPH (×10^9/L). All data were independently cross-checked by two investigators.

### 2.5 Statistical analysis

For continuous variables, values are presented as mean ± SD when distributions were approximately normal; otherwise, median (Q1–Q3) is reported. The independent-samples t-test was used for two-group comparisons of normally distributed variables, while the Mann–Whitney U test was applied for non-normally distributed data. Categorical variables and proportions were compared using the Chi-square test. Differences among the anti-dsDNA(-), anti-dsDNA-L, and anti-dsDNA-H groups were analyzed using the Kruskal–Wallis H test, followed by Dunn’s *post hoc* test for pairwise comparisons. Associations between anti-dsDNA and other laboratory indices were evaluated using Spearman’s rank correlation. Receiver operating characteristic (ROC) curves were used to evaluate the discriminative performance of PNI, the CD4^+^/CD8^+^ ratio, and 25(OH)D, individually and in combination, for anti-dsDNA-based stratification. Specifically, ROC analyses were performed for two binary outcomes: anti-dsDNA(+) versus anti-dsDNA(-), and anti-dsDNA-H versus anti-dsDNA-L. The combined model was constructed using binary logistic regression based on three pre-specified biomarkers, namely 25(OH)D, the CD4^+^/CD8^+^ ratio, and PNI, whereas C3 was additionally analyzed as a benchmark comparator because of its established role in routine SLE assessment. Optimal cutoff values were determined using the Youden index, and pairwise differences between AUCs were compared using the DeLong test. Ordinal logistic regression was performed to identify factors independently associated with anti-dsDNA titer strata. The proportional odds assumption was assessed, and collinearity was evaluated using the variance inflation factor (VIF). No significant multicollinearity was observed (all VIF < 5). Results are presented as odds ratios (ORs) with 95% confidence intervals (CIs). To assess the robustness of the findings, sensitivity analysis was performed by excluding patients with renal injury and removing those with a 1:32 anti-dsDNA titer. Ordinal logistic regression was used in both cases to evaluate the associations. Additionally, a binary logistic regression model was applied to generate predicted probabilities for the combined indicators, which were subsequently used in ROC curve analysis. All tests were two-sided, and P < 0.05 was considered statistically significant. Statistical analyses were performed using IBM SPSS Statistics (version 26.0) and GraphPad Prism (version 10.0).

## 3 Results

### 3.1 Baseline and core indicators comparison in SLE patients with various anti-dsDNA status

Among the 126 patients, 64 were anti-dsDNA(-) and 62 were anti-dsDNA(+); among the anti-dsDNA(+) patients, 16 had a titer of 1:10, 21 had a titer of 1:32, and 25 had titers ≥1:100. The comparison of baseline characteristics and core indicators between the anti-dsDNA(-) and anti-dsDNA(+) groups is shown in **Table 1**. Compared with the anti-dsDNA(-) group, the anti-dsDNA(+) group had significantly lower levels of complement 3 (C3), complement 4 (C4), CD4^+^/CD8^+^ ratio, 25-hydroxyvitamin D, and prognostic nutritional index (PNI), as well as a higher incidence of renal injury (all P < 0.05). The subgroup analysis stratified by anti-dsDNA titer is presented in **Table 2**. Patients in the high-titer group exhibited significantly lower levels of C3, C4, and 25-hydroxyvitamin D, as well as a significantly reduced CD4^+^/CD8^+^ ratio compared with those in the low-titer group (all P < 0.05). Although PNI levels showed a decreasing trend in the high-titer group, the difference was not statistically significant (P > 0.05).

**Table 1:** Comprehensive demographic, clinical, and laboratory characteristics of included patients. .

Characteristics	Anti-dsDNA(-) (n = 64)	Anti-dsDNA(+) (n = 62)	χ²/Z/t	P
Sex (male/female)	8/56	7/55	0.044	0.834
Age (year)	45.92 ± 15.45	40.15 ± 16.53	2.028	0.045
Disease Course (year)	3.00 (0.85-9.00)	1.00 (0.10-6.25)	-2.643	0.008
ALB (g/L)	39.40 (35.40-42.05)	36.40 (31.48-39.32)	-3.272	0.001
C3 (g/L)	0.97 ± 0.31	0.67 ± 0.28	5.654	0.001
C4 (g/L)	0.13 (0.09-0.21)	0.09(0.03-0.12)	-3.992	<.001
CRP (mg/L)	3.13 (0.66-19.72)	5.17(1.63-12.76)	-1.022	0.307
ESR (mm/h)	29.50 (10.25-59.00)	48.50 (26.00-77.75)	-2.955	0.003
NEUT (× 10^9^/L)	3.49 (2.09-5.49)	3.49 (1.90-4.79)	-0.537	0.591
LYMPH (× 10^9^/L)	1.04 (0.82-1.42)	0.76 (0.60-1.27)	-2.750	0.006
PLT (× 10^9^/L)	198.00 (153.75-233.50)	205.50 (154.00-251.25)	-0.656	0.512
Total CD3+ (%)	74.64 (68.16-81.78)	74.11 (64.58-83.05)	-0.110	0.913
CD3+CD4+ (%)	36.59 (29.53-43.23)	31.02 (25.34-40.39)	-2.44	0.015
CD4^+^/CD8^+^	1.09 (0.83-1.71)	0.91 (0.68-1.23)	-2.543	0.011
CD16^+^CD56^+^ (%)	8.48 (6.02-13.35)	8.94 (5.10-13.36)	-0.447	0.655
CD19^+^ B (%)	12.50 (6.64-19.38)	14.40 (9.77-23.14)	-1.391	0.164
PNI	45.00 (41.65-48.33)	40.73 (34.95-45.74)	-3.853	<0.001
25(OH)D (ng/mL)	17.14 (13.96-22.49)	12.54 (9.68-16.79)	-4.992	<0.001
Anti-Sm, n (%)	27/42.19	25/40.32	0.045	0.832
Anti-SSA, n (%)	27/42.19	33/53.23	1.538	0.215
Anti-SSB, n (%)	13/20.31	20/32.26	2.795	0.095
Hematologic involvement, n (%)	28/43.75	33/53.23	1.689	0.194
Renal involvement, n (%)	19/29.69	33/53.23	8.276	0.004

Data are presented as mean ± SD or median (Q1–Q3). Categorical data are shown as n (%) Abbreviations: SD, standard deviation; Q1, first quartile; Q3, third quartile. ALB, albumin; C3, complement 3; C4, complement 4; CRP, C-reactive protein; ESR, erythrocyte sedimentation rate; NEUT, neutrophil; LYMPH, lymphocyte; PLT, platelet; PNI, prognostic nutritional index; 25(OH)D, 25-hydroxyvitamin D.

**Table 2:** Core immunological and nutritional indicators stratified by anti-dsDNA antibody titer levels.

Characteristics	Anti-dsDNA-L (n = 37)	Anti-dsDNA-H (n = 25)	χ²/Z/t	*P*
Sex (male/female)	3/34	4/21	0.928	0.425
Age (year)	40.43 ± 16.38	39.72 ± 17.08	0.164	0.871
Disease Course	1.00 (0.15-6.50)	0.50 (0.10-6.50)	-0.58	0.562
PNI	43.70 (35.75-46.1)	37.55 (34.13-42.8)	-1.901	0.057
25(OH)D (ng/mL)	15.38 (10.85-17.51)	10.95 (9.18-13.15)	-2.813	0.005
CD4^+^/CD8^+^	1.04 (0.75-1.56)	0.74 (0.43-1.05)	-2.935	0.003
CRP (mg/L)	5.21 (1.77-31.95)	5.12 (1.43-10.27)	-1.033	0.302
ESR (mm/h)	44.00(21.50-78.00)	53.00 (34.50-79.50)	-1.199	0.231
C3 (g/L)	0.75 ± 0.27	0.56 ± 0.25	2.868	0.006
C4 (g/L)	0.10 (0.05-0.15)	0.04 (0.02-0.10)	-2.332	0.020
Anti-Sm, n (%)	16/43.24	9/36.00	0.568	0.325
Anti-SSA, n (%)	21/56.76	12/48.00	0.460	0.498
Anti-SSB, n (%)	13/35.14	7/28.00	0.348	0.555
Hematologic involvement, n (%)	17/45.95	16/64.00	1.953	0.162
Renal involvement, n (%)	16/43.24	17/68.00	3.673	0.055

Data are presented as mean ± SD or median (Q1–Q3). Categorical data are shown as n (%). Abbreviations: SD, standard deviation; Q1, first quartile; Q3, third quartile. ALB, albumin; C3, complement 3; C4, complement 4; CRP, C-reactive protein; ESR, erythrocyte sedimentation rate; NEUT, neutrophil; LYMPH, lymphocyte; PLT, platelet; PNI, prognostic nutritional index; 25(OH)D, 25-hydroxyvitamin D. anti-dsDNA-L, low-titer anti-dsDNA group; anti-dsDNA-H, high-titer anti-dsDNA group. Anti-dsDNA positivity was defined as a titer ≥1:10. The low-titer group included patients with titers of 1:10 (n=16) and 1:32 (n=21); the high-titer group included those with titers ≥1:100 (n=25).

### 3.2 Comparison of PNI, CD4^+^/CD8^+^ ratio, and 25(OH)D levels in SLE patients stratified by anti-dsDNA titer

**Figure 2:**
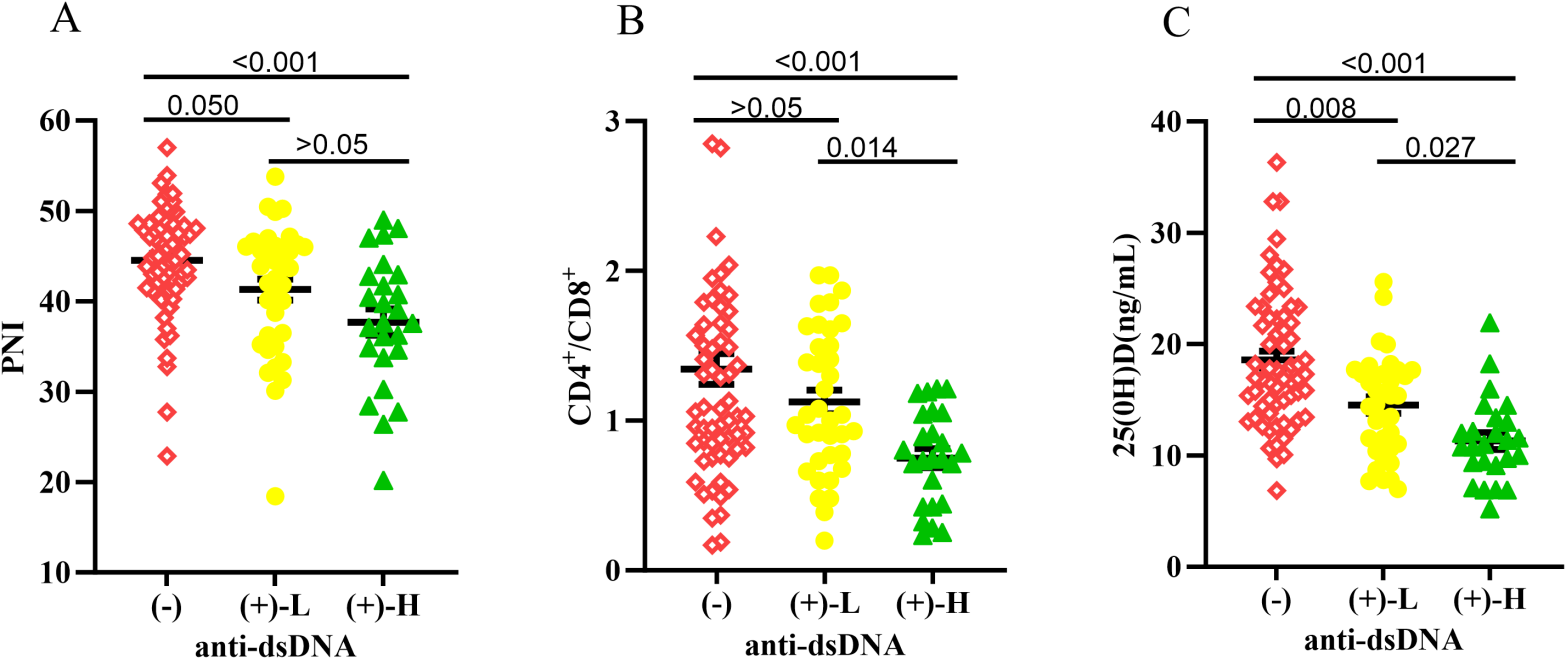
Scatter plots showing **(A)** PNI, **(B)** CD4^+^/CD8^+^ ratio, and **(C)** 25(OH)D levels across anti-dsDNA antibody groups: negative (-), high-titer positive (+)-H, and low-titer positive (+)-L. Each dot represents an individual participant, and horizontal lines indicate the median values. Differences among groups were analyzed using the Kruskal–Wallis test with Dunn’s *post hoc* test.

As shown in **Figure 2**, PNI, the CD4^+^/CD8^+^ ratio, and 25(OH)D differed significantly among the three groups. *Post hoc* analysis showed that PNI was significantly lower in both anti-dsDNA(+) groups than in the anti-dsDNA(-) group, with no significant difference between the anti-dsDNA-L and anti-dsDNA-H groups. The anti-dsDNA-H group had a significantly lower CD4^+^/CD8^+^ ratio than the other two groups, while 25(OH)D differed significantly across all pairwise comparisons.

### 3.3 Correlation matrix of 25(OH)D, PNI, CD4^+^/CD8^+^ ratio and anti-dsDNA in patients with SLE

**Figure 3:**
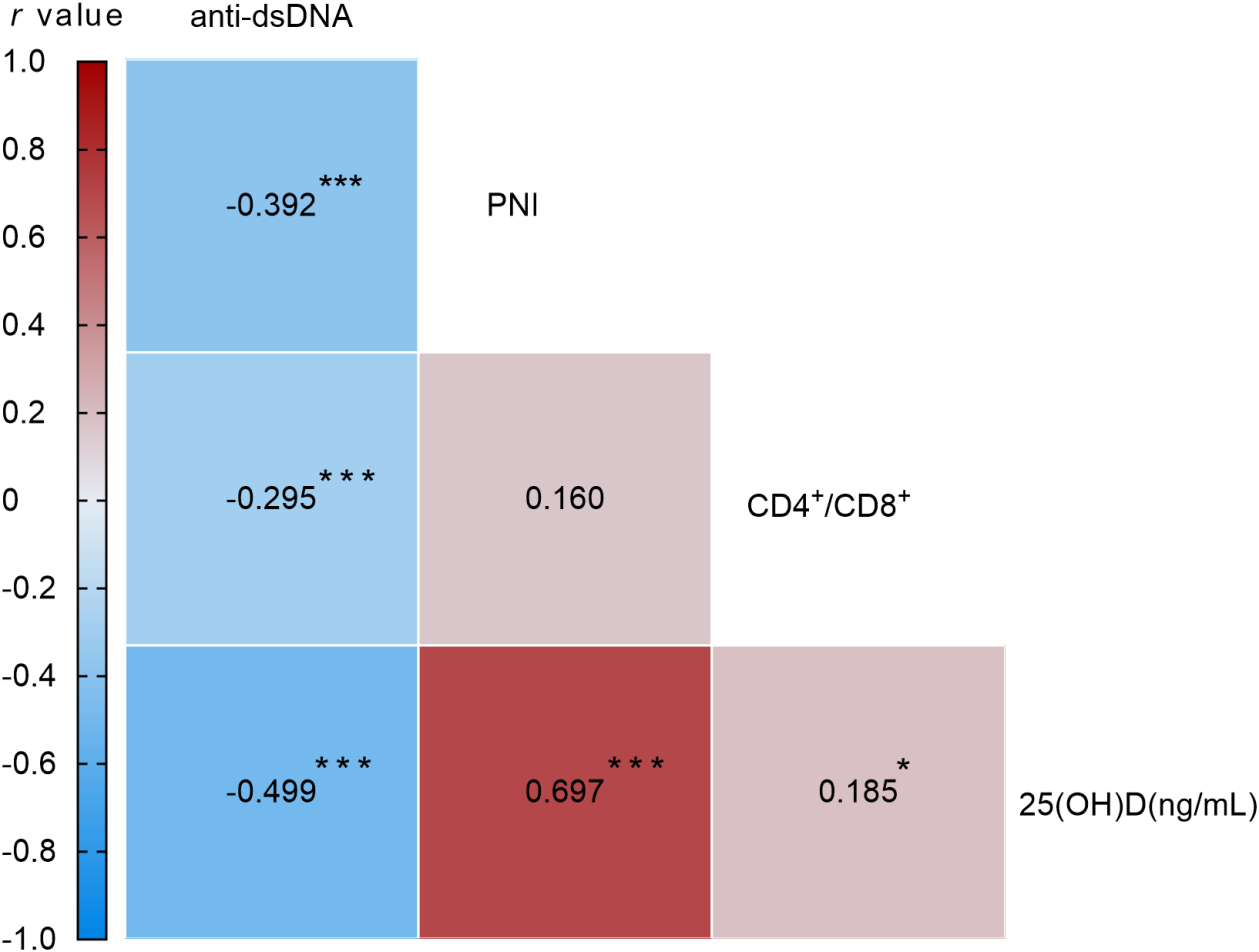
Correlation matrix of 25(OH)D, PNI, CD4^+^/CD8^+^, and anti-dsDNA in patients with SLE. Values are Spearman correlation coefficients. *p < 0.05, **p < 0.001, ***p < 0.001.

Spearman correlation analysis revealed that anti-dsDNA antibody levels were significantly and negatively correlated with 25(OH)D (r = -0.499, *P* < 0.001), PNI (r = -0.392, *P* < 0.001), and the CD4^+^/CD8^+^ ratio (r = -0.295, *P* < 0.001) (**Figure 3**).

### 3.4 ROC analysis for anti-dsDNA-based stratification in patients with SLE

**Figure 4:**
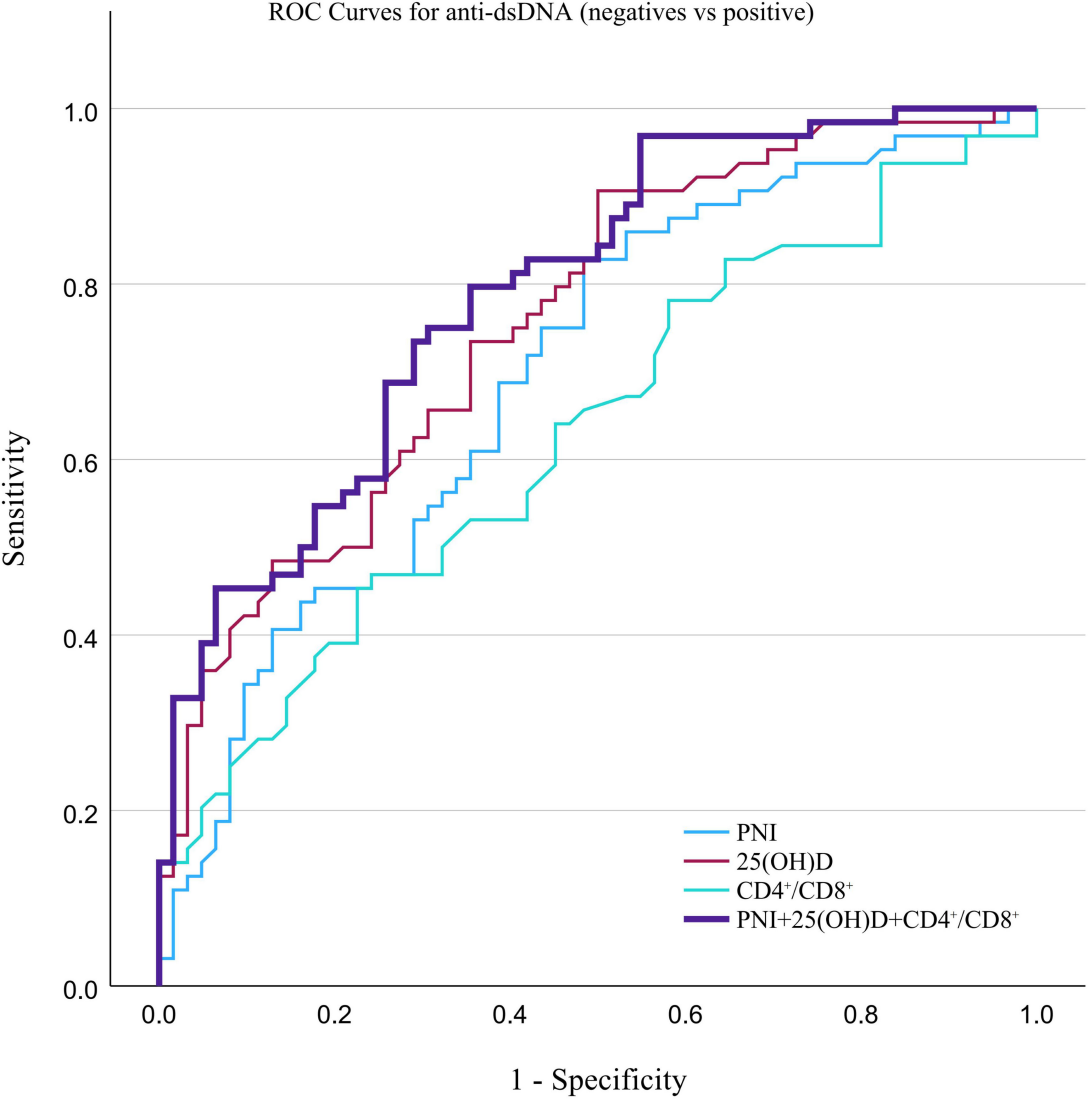
Receiver operating characteristic (ROC) curve analysis evaluating the discriminatory performance of the CD4^+^/CD8^+^ ratio, 25(OH)D, and PNI for stratifying anti-dsDNA(-) and anti-dsDNA(+) status. The AUC, area under the curve and corresponding 95% confidence intervals are provided in **Table 3**.

**Figure 5:**
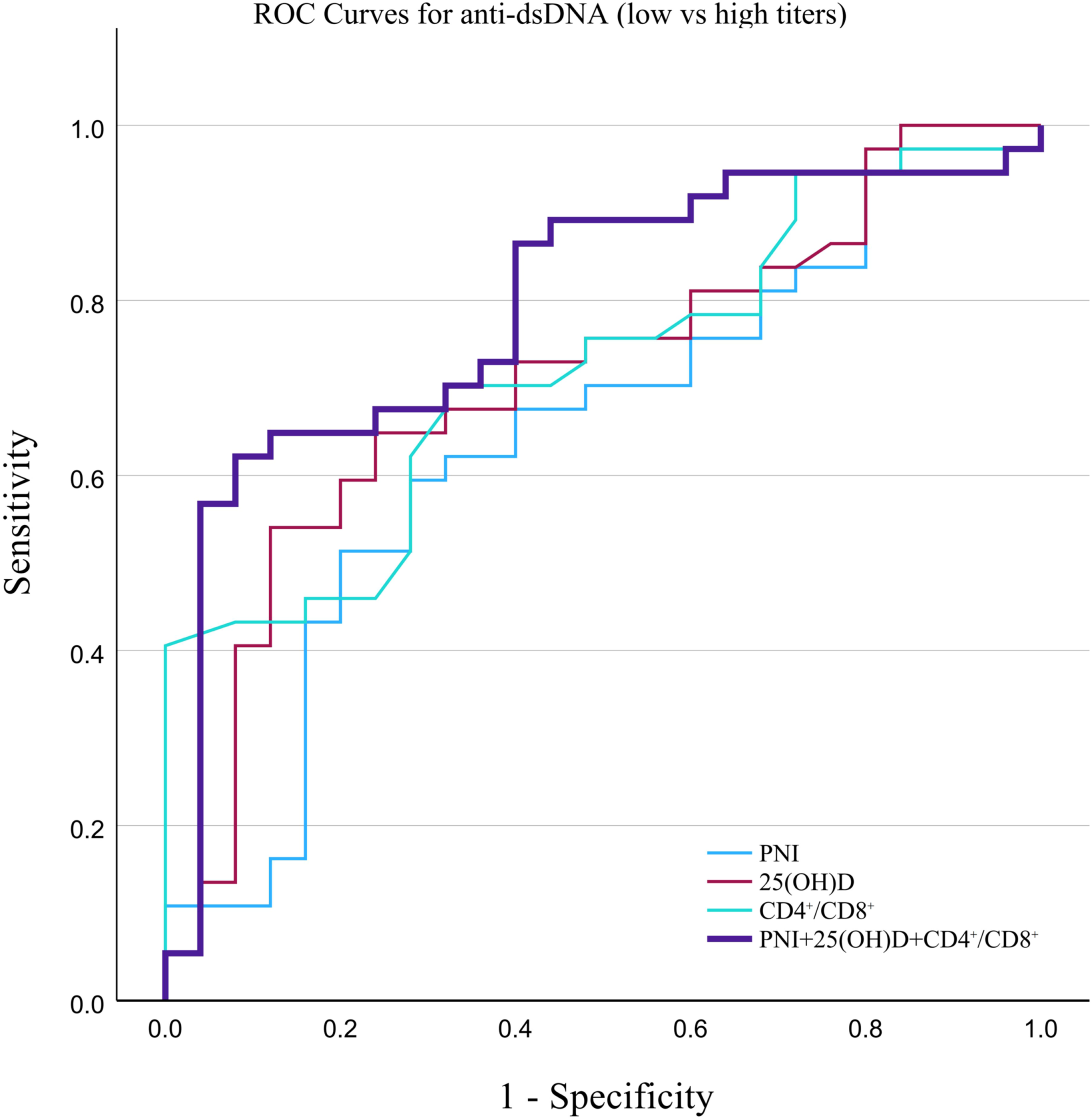
Receiver operating characteristic (ROC) curve analysis evaluating the discriminatory performance of the CD4^+^/CD8^+^ ratio, 25(OH)D, and PNI for stratifying anti-dsDNA-L and anti-dsDNA-H groups. The AUC, area under the curve and corresponding 95% confidence intervals are provided in **Table 4**.

The combined model was constructed using binary logistic regression based on three pre-specified biomarkers: 25(OH)D, the CD4^+^/CD8^+^ ratio, and PNI. The results of the binary logistic regression model, including odds ratios (ORs) for each biomarker, are shown in **Supplementary Tables S1**, **S2**. These predicted probabilities were then applied in the ROC curve analysis to evaluate the discriminatory performance of the combined model for anti-dsDNA-based stratification. Specifically, ROC curve analysis showed that, for stratifying anti-dsDNA(+) from anti-dsDNA(-) patients, 25(OH)D had the highest AUC among the individual markers (0.758), followed by PNI (0.699) and CD4^+^/CD8^+^ ratio (0.631), while the combined model achieved the highest AUC of 0.792 (**Table 3**; **Figure 4**). For stratifying low and high titer anti-dsDNA groups, the CD4^+^/CD8^+^ ratio showed the highest individual AUC (0.721), followed by 25(OH)D (0.712) and PNI (0.643), whereas the combined model achieved the highest AUC of 0.795 (**Table 4**; **Figure 5**).

**Table 3:** ROC analysis for stratifying anti-dsDNA positivity in patients with SLE.

Marker/model	AUC (95% CI)	*p*	Cut-off value	Sensitivity (%)	Specificity (%)
C3 (g/L)	0.751(0.676-0.841)	<0.001	0.821	69.40	70.30
25(OH)D (ng/mL)	0.758 (0.675–0.841)	<0.001	12.19	90.60	50.00
CD4^+^/CD8^+^	0.631(0.534–0.728)	0.008	1.305	45.30	73.40
PNI	0.699 (0.607–0.790)	<0.001	40.90	48.40	65.60
PNI + 25(OH)D + CD4^+^/CD8^+^	0.792(0.715–0.869)	<0.001	—	73.40	71.00

AUC, area under the curve; CI, confidence interval; PNI, prognostic nutritional index; 25(OH)D, 25-hydroxyvitamin D; CD4^+^/CD8^+^, CD4^+^/CD8^+^ ratio. The optimal cut-off value was determined by maximizing the Youden’s index.

**Table 4:** ROC analysis for stratifying high anti-dsDNA titer among anti-dsDNA(+) patients with SLE.

Marker/model	AUC (95% CI)	*p*	Cut-off value	Sensitivity (%)	Specificity (%)
C3 (g/L)	0.631(0.485-0.777)	0.079	0.655	64.00	64.90
25(OH)D (ng/mL)	0.712 (0.579–0.844)	0.001	14.54	54.10	88.00
CD4^+^/CD8^+^	0.721(0.595–0.847)	0.002	1.255	40.50	100.00
PNI	0.643(0.500–0.786)	0.050	40.50	59.50	72.00
PNI + 25(OH)D + CD4^+^/CD8^+^	0.795(0.678–0.911)	<0.001	—	62.20	92.00

AUC, area under the curve; CI, confidence interval; PNI, prognostic nutritional index; 25(OH)D, 25-hydroxyvitamin D; CD4^+^/CD8^+^, CD4^+^/CD8^+^ ratio. The optimal cut-off value was determined by maximizing the Youden’s index.

For the comparison between anti-dsDNA(-) and anti-dsDNA(+) groups, C3 showed only marginal predictive value, and the combined indicator exhibited significantly better discrimination than both C3 and PNI. For the negative-versus-positive comparison, the combined indicator outperformed PNI and the CD4^+^/CD8^+^ ratio, but offered no significant advantage over C3 and 25(OH)D (DeLong test, *P* > 0.05).

### 3.5 Indicators associated with anti-dsDNA antibody grade in SLE patients: Ordinal logistic regression analysis.

The anti-dsDNA antibody grade was categorized into three levels based on antibody titers: anti-dsDNA(-), anti-dsDNA-L, and anti-dsDNA-H. To identify indicators associated with anti-dsDNA antibody grade, univariate and multivariate ordinal logistic regression analyses were performed (**Table 5**). Univariate ordinal logistic regression analysis showed that disease course, C3, CD4^+^/CD8^+^ ratio, 25(OH)D, and PNI were significantly associated with anti-dsDNA antibody grade. Age showed a marginal association. In the multivariate analysis, after adjusting for confounding factors, C3 and 25(OH)D remained independently associated with higher anti-dsDNA antibody grade. Other variables, including disease course, CD4^+^/CD8^+^ ratio, and PNI, were no longer statistically significant. The sensitivity analysis, which excluded patients with renal injury and those with a 1:32 anti-dsDNA titer, was performed using ordinal logistic regression. The findings of this analysis are shown in **Supplementary Table S3**, and they did not significantly alter the overall results.

**Table 5:** Univariate and multivariate ordinal logistic regression analyses for anti-dsDNA antibody grade in patients with SLE.

Project	Univariate OR (95% CI)	*p*	Multivariable OR (95% CI)	*p*
Age (year)	0.979 (0.959–1.000)	0.052	--	--
Disease Course	0.936 (0.876–0.999)	0.048	0.971 (0.901–1.048)	0.459
C3 (g/L)	0.035 (0.009–0.132)	<0.001	0.110 (0.024–0.499)	0.004
CD4^+^/CD8^+^	0.317 (0.163–0.613)	<0.001	0.516 (0.257–1.038)	0.063
25(OH)D (ng/mL)	0.804 (0.741–0.874)	<0.001	0.821 (0.738–0.913)	<.001
PNI	0.898 (0.852–0.946)	<0.001	1.009 (0.933–1.092)	0.815

OR, adjusted odds ratio; 95% CI, 95% confidence interval. Multivariate model was adjusted for all variables included in the table. Parallel lines test *P* = 0.105, Nagelkerke *R²* = 0.437.

## 4 Discussion

SLE is a highly heterogeneous autoimmune disease, and a single serological marker is often insufficient to comprehensively capture its complex immunopathological profile. Although anti-dsDNA antibodies have long been recognized as an important biomarker for monitoring disease activity in SLE, previous studies have mainly focused on their association with overall disease activity and renal involvement (7, 19). By contrast, the characteristics of nutritional status, vitamin D metabolism, and cellular immune imbalance across different anti-dsDNA titer categories have not been fully elucidated. In the present study, patients were stratified into anti-dsDNA(-), anti-dsDNA-L, and anti-dsDNA-H groups according to antibody titers, and three readily available indicators were jointly evaluated, namely 25(OH)D, CD4^+^/CD8^+^ ratio, and PNI. This multidimensional framework, integrating vitamin-related, cellular immune, and immunonutritional parameters, may better reflect the underlying nature of SLE, in which humoral immune abnormalities, cellular immune dysregulation, and chronic inflammatory/nutritional impairment coexist. Therefore, this anti-dsDNA titer-based multidimensional assessment strategy may help overcome the limitations of conventional single-marker monitoring and provide a more practical basis for refined clinical stratification of patients with SLE.

This study showed that 25(OH)D levels differed significantly among the anti-dsDNA(-), anti-dsDNA-L, and anti-dsDNA-H groups, and all pairwise comparisons remained significant after Bonferroni correction. In ordinal regression analysis, 25(OH)D was also independently associated with anti-dsDNA status. These findings indicate that 25(OH)D levels decreased progressively with increasing anti-dsDNA titers, suggesting that 25(OH)D was the most sensitive of the three indicators for stratifying anti-dsDNA titer strata. This result is consistent with previous studies showing that low vitamin D levels are associated with increased susceptibility to SLE and greater disease severity, whereas vitamin D supplementation is associated with a modest but statistically significant reduction in disease activity scores (20–22). Previous studies have also suggested that vitamin D is not only a nutritional marker in SLE, but also an important immunomodulatory factor involved in the maintenance of Treg homeostasis, suppression of Th17-related inflammation, and regulation of abnormal B-cell activation (23). From a mechanistic perspective, an increase in anti-dsDNA titers usually reflects enhanced autoantibody production, more active humoral immune dysregulation, and more pronounced formation and deposition of immune complexes, whereas a decrease in 25(OH)D levels may indicate impaired regulation of abnormal immune activation by the host (24–26). As an important molecule with both endocrine and immunomodulatory functions, vitamin D contributes to immune homeostasis in SLE by influencing T-cell differentiation, B-cell activation, and inflammatory cytokine expression (22). In the present study, 25(OH)D not only stratified anti-dsDNA(-) from anti-dsDNA(+) patients, but also further differentiated low-titer from high-titer positive cases, indicating that it has a good gradient-reflecting ability for serum anti-dsDNA levels in SLE. In other words, 25(OH)D appears to function more as a dynamic biomarker that declines with progressive immune dysregulation rather than as a simple nutritional accompaniment.

In contrast to 25(OH)D, the CD4^+^/CD8^+^ ratio was comparable between anti−dsDNA−negative and low−titer positive individuals, while obvious differences emerged when comparing the negative group with the high−titer group, as well as between low−titer and high−titer subgroups. This distribution pattern implies that the CD4^+^/CD8^+^ ratio is insensitive to mild early serological abnormalities in SLE, and primarily reflects immune dysregulation occurring alongside marked increases in autoantibody titers. Recent studies on T-cell abnormalities in SLE have shown that patients with active disease often exhibit decreased peripheral CD3+ and CD4+ T-cell counts, together with abnormally increased expression of activation markers such as CD38 (27–29). Other studies have reported that the abnormal expansion of cytotoxic effector CD4^+^/CD8^+^T-cell subsets is closely associated with disease activity and tissue damage in SLE (30, 31). Correlation heat map analysis confirmed a definite association between the CD4^+^/CD8^+^ ratio and anti−dsDNA titers. However, such a correlation did not translate into a consistent stepwise trend in ordinal regression, which was largely due to the lack of distinction between the negative and low-titer groups. Thus, the CD4^+^/CD8^+^ ratio showed a closer association with high-titer anti-dsDNA levels than with mild or low-level serological abnormalities, supporting its auxiliary value in identifying more advanced immune alterations linked to high-titer anti-dsDNA status in SLE.

PNI showed an overall significant difference among the three groups; however, *post hoc* pairwise comparisons indicated that PNI was relatively sensitive to the transition from anti-dsDNA(-) to anti-dsDNA(+) status, whereas its ability to further stratify between different positive titer levels was limited. Calculated from serum albumin concentration and peripheral lymphocyte count, PNI essentially reflects the combined status of inflammatory burden, nutritional reserve, and immune function. Recent studies have shown that PNI is associated with disease activity in SLE, and that its ability to identify active SLE can be further improved when combined with indicators such as the fibrinogen-to-albumin ratio (32). In addition, malnutrition is relatively common among hospitalized patients with SLE and is closely associated with disease activity, renal involvement, lymphopenia, and low complement levels (33). In the present study, no significant difference in PNI was observed between the low-titer and high-titer groups, suggesting that once patients enter the anti-dsDNA(+) state, the nutrition–inflammation axis may already be substantially affected, and further increases in antibody titers may not necessarily be accompanied by a parallel linear decline in PNI. This may be explained by the fact that serum albumin is influenced by multiple factors, including renal loss, inflammatory responses, hepatic synthetic function, and treatment-related effects. Therefore, PNI may serve as a useful indicator for identifying prominent immune-inflammatory changes linked to heightened disease activity, rather than a precise marker that varies in parallel with anti-dsDNA titers. Given the non−significant difference between low− and high−titer subgroups, this pattern may reflect limited statistical power, rather than a clear gradation corresponding to antibody levels.

ROC analysis further supported the above findings. For discrimination between anti-dsDNA(-) and anti-dsDNA(+) patients, 25(OH)D showed the highest AUC among the individual biomarkers, indicating the best standalone discriminatory performance. PNI showed moderate discrimination, whereas the CD4^+^/CD8^+^ ratio performed less well in this binary setting. The combined assessment of 25(OH)D, the CD4^+^/CD8^+^ ratio, and PNI achieved the highest overall AUC. Although its AUC was not significantly different from that of C3, the combined assessment showed a more balanced sensitivity and specificity profile, suggesting that these biomarkers may provide complementary information for anti-dsDNA serostatus stratification.

The advantage of the combined assessment was more evident in stratifying low-positive from high-positive anti-dsDNA titers. DeLong testing showed that the combined assessment had a significantly higher AUC than PNI and C3, indicating better discriminatory performance in this setting. Among the individual biomarkers, the CD4^+^/CD8^+^ ratio and 25(OH)D both showed higher AUCs than PNI, which was consistent with the group comparison results. Overall, these findings suggest that the combined assessment may provide additional stratification information beyond routine complement assessment, particularly for differentiating anti-dsDNA titer levels. However, these results should still be interpreted as exploratory and require validation in larger external cohorts.

The present study has several limitations. First, as a single-center cross-sectional study, it identified associations between anti-dsDNA titers and 25(OH)D, the CD4+/CD8+ ratio, and PNI, but could not establish causal relationships. Second, the levels of these biomarkers may be influenced by factors other than SLE itself, such as season, sunlight exposure, renal function, and vitamin D supplementation. Third, the relatively small sample size of the anti-dsDNA(+) group (n = 25) may limit statistical power, potentially leading to type II errors. Additionally, these biomarkers were not evaluated alongside validated disease activity measures, and their clinical relevance beyond anti-dsDNA-based stratification requires further confirmation in larger prospective studies. Finally, the anti-dsDNA titer grouping was based on the local laboratory assay system, which may limit the generalizability of the findings to other centers using different testing platforms or thresholds.

## 5 Conclusion

In summary, this study evaluated the potential value of 25(OH)D, the CD4^+^/CD8^+^ ratio, and PNI for anti-dsDNA-based stratification in patients with SLE. The combined assessment demonstrated the highest AUC for stratifying anti-dsDNA(-) from anti-dsDNA(+) patients, with performance comparable to C3. More importantly, it showed significantly better discriminatory performance than C3 in stratifying low-positive from high-positive anti-dsDNA titers, suggesting its potential for more refined serological stratification. Among the individual biomarkers, 25(OH)D showed relatively stable discriminatory ability in both anti-dsDNA serostatus and titer stratification, while the CD4^+^/CD8^+^ ratio showed better value in differentiating titer levels. PNI may provide complementary information from a nutritional-inflammatory perspective. Overall, the combined assessment integrates information on vitamin D metabolism, cellular immunity, and nutritional status, offering additional stratification value beyond routine complement assessment. However, given the single-center cross-sectional design, these findings should be interpreted as exploratory and require validation in larger external cohorts.

## Data Availability

The raw data supporting the conclusions of this article will be made available by the authors, without undue reservation.
