# Supplementary material for: Combined assessment of 25(OH)D, CD4+/CD8+ ratio, and prognostic nutritional index for anti-dsDNA-based stratification in systemic lupus erythematosus
Source: Front Immunol. 2026 Apr 29;17:1841620. doi: 10.3389/fimmu.2026.1841620 (PMC13167970; doi:10.3389/fimmu.2026.1841620)
Supplement: Supplementary file 1 [file Table1.docx]

## Table S1. Multivariable binary logistic regression model for the combination of PNI, 25(OH)D, and CD4^+^/CD8+ ratio in distinguishing anti-dsDNA(-) and anti-dsDNA(+) status

| Variable | B | SE | Wald χ² | P | OR (95% CI) |
| --- | --- | --- | --- | --- | --- |
| 25(OH)D(ng/mL) | -0.164 | 0.050 | 10.731 | 0.001 | 0.849 (0.770–0.936) |
| CD4^+^/CD8^+^ | -0.827 | 0.353 | 5.488 | 0.019 | 0.437 (0.219–0.874) |
| PNI | -0.043 | 0.041 | 1.144 | 0.285 | 0.958 (0.884–1.037) |
| Constant | 5.350 | 1.592 | 11.297 | <0.001 | - |

**Note:** A multivariable binary logistic regression model was constructed with anti-dsDNA status as the binary dependent variable, and PNI, 25(OH)D, and CD4^+^/CD8^+^ ratio as independent variables. The model was statistically significant (Omnibus χ² = 37.263, df = 3, *P* < 0.001), with satisfactory calibration (Hosmer–Lemeshow χ² = 2.941, df = 8, *P* = 0.938) and a Nagelkerke R² of 0.341 for discriminative ability.

**Table S2.** Multivariable binary logistic regression model for the combination of PNI, 25(OH)D, and CD4+/CD8+ ratio in distinguishing anti-dsDNA-L and anti-dsDNA-H status

| Variable | B | SE | Wald χ² | P | OR (95% CI) |
| --- | --- | --- | --- | --- | --- |
| 25(OH)D(ng/mL) | **-0.240** | **0.090** | **7.160** | **0.007** | **0.786 (0.659–0.938)** |
| **CD4^+^/CD8^+^** | **-1.646** | **0.625** | **6.941** | **0.008** | **0.193 (0.057–0.656)** |
| **PNI** | **-0.002** | **0.052** | **0.001** | **0.975** | **0.998 (0.901–1.106)** |
| **Constant** | **3.619** | **1.667** | **4.713** | **0.030** | **-** |

**Note: A** multivariable binary logistic regression model was constructed with anti-dsDNA status (negative vs. positive) as the binary dependent variable, and PNI, 25(OH)D, and CD4^+^/CD8^+^ ratio as independent predictors in the (high/low) subgroup. The model was statistically significant (Omnibus χ² = 35.335, df = 3, *P* < 0.001), with satisfactory calibration (Hosmer–Lemeshow χ² = 7.388, df = 8, *P* = 0.495) and a Nagelkerke R² of 0.388 for discriminative ability.

**Table S3.** Sensitivity analyses for anti‑dsDNA antibody grade in SLE patients: multivariate ordinal logistic regression

| **Variable** | Patients with renal involvement were excluded | | Patients with anti-dsDNA titer 1:32 were excluded | |
| --- | --- | --- | --- | --- |
|  | OR (95%CI) | *P* | OR (95%CI) | *P* |
| Disease Course | 1.038 (0.935–1.150) | 0.489 | 0.990 (0.907–1.081) | 0.830 |
| C3 (g/L) | 0.051 (0.006–0.446) | 0.007 | 0.096 (0.016–0.576) | 0.011 |
| 25(OH)D(ng/mL) | 0.807 (0.689–0.944) | 0.007 | 0.829 (0.735–0.936) | 0.002 |
| PNI | 1.041 (0.907–1.195) | 0.572 | 0.964 (0.872–1.064) | 0.464 |
| **CD4+/CD8+** | 0.554 (0.241–1.270) | 0.163 | 0.471 (0.204–1.092) | 0.079 |

**Note:** Left columns: Patients with lupus nephritis were excluded (parallel lines test P=0.139, Nagelkerke R²=0.362). Right columns: Patients with anti-dsDNA titer 1:32 were excluded (parallel lines test P = 0.348, Nagelkerke R² = 0.468). All models satisfied the proportional odds assumption. OR, adjusted odds ratio; 95%CI, 95% confidence interval.
